# Supplementary material for: Mechanisms governing the pioneering and redistribution capabilities of the non-classical pioneer PU.1
Source: Nat Commun. 2020 Jan 21;11:402. doi: 10.1038/s41467-019-13960-2 (PMC6972792; doi:10.1038/s41467-019-13960-2)
Supplement: Supplementary file 7 — Source data [file 41467_2019_13960_MOESM7_ESM.zip › Source_Data/FigureS2/FigureS2B_MotifScanOutput/KmeansCluster2/homerResults/motif36.similar.html]

motif36

## Information for motif36

C
G
T
A
A
G
T
C
A
C
G
T
A
G
T
C
C
T
G
A
A
G
T
C
A
C
G
T
C
T
G
A
A
G
C
T
A
G
T
C
  
Reverse Opposite:  

C
T
A
G
C
T
G
A
A
G
C
T
C
G
T
A
A
C
T
G
A
G
C
T
A
C
T
G
C
G
T
A
A
C
T
G
A
C
G
T
  

|  |  |
| --- | --- |
| p-value: | 1e-28 |
| log p-value: | -6.615e+01 |
| Information Content per bp: | 1.935 |
| Number of Target Sequences with motif | 86.0 |
| Percentage of Target Sequences with motif | 1.71% |
| Number of Background Sequences with motif | 122.2 |
| Percentage of Background Sequences with motif | 0.30% |
| Average Position of motif in Targets | 99.9 +/- 49.3bp |
| Average Position of motif in Background | 89.8 +/- 73.1bp |
| Strand Bias (log2 ratio + to - strand density) | -0.1 |
| Multiplicity (# of sites on avg that occur together) | 1.02 |
| Motif File: | file (matrix) reverse opposite |

### Similar de novo motifs found

|  |  |  |  |  |  |  |  |
| --- | --- | --- | --- | --- | --- | --- | --- |
| Rank | Match Score | Redundant Motif | P-value | log P-value | % of Targets | % of Background | Motif file |
| 1 | 0.839 | C T G A A G T C C G A T A G T C C T A G A G T C A C G T C T G A A C G T | 1e-25 | -58.430249 | 1.47% | 0.25% | motif file (matrix) |
| 2 | 0.782 | A G T C A C G T G A T C C G T A A G T C A C G T A G T C C T G A A G T C A G C T C G T A | 1e-23 | -54.952037 | 1.27% | 0.20% | motif file (matrix) |
| 3 | 0.778 | T G C A A G T C A C G T A G T C C T A G G T A C A C G T C G T A | 1e-15 | -35.075228 | 2.19% | 0.84% | motif file (matrix) |
